# Nickel-Based Selenides with a Fractal Structure as an Excellent Bifunctional Electrocatalyst for Water Splitting

**DOI:** 10.3390/nano12020281

**Published:** 2022-01-17

**Authors:** Jingxuan He, Ting Qian, Chao Cai, Xia Xiang, Sean Li, Xiaotao Zu

**Affiliations:** 1 Yangtze Delta Region Institute (Huzhou), University of Electronic Science and Technology of China, Huzhou 313001, China; hejx10@qq.com (J.H.); 1530554402@qq.com (T.Q.); 2 School of Physics, University of Electronic Science and Technology of China, Chengdu 611731, China; xiaxiang@uestc.edu.cn; 3 School of Materials Science and Engineering, The University of New South Wales, Sydney 2052, Australia

**Keywords:** electrochemical, HER, OER, nickel selenide, doping, electrocatalytic water splitting

## Abstract

Nickel-based selenides are believed to be promising non-precious metal electrocatalysts, and have been widely used for both oxygen evolution reactions (OER) and hydrogen evolution reactions (HER). Here, we control the aging time to prepare Ni_x_Se_y_ with different fractal structures as a bifunctional catalyst. An obtained sample with an aging time of 80 min shows outstanding electrocatalytic performance for hydrogen evolution reactions (HER) with an overpotential of 225 mV (η@10 mA/cm^2^) and for oxygen evolution reactions (OER) with an overpotential of 309 mV (η@50 mA/cm^2^). Moreover, to further improve catalytic activity, we doped Fe in Ni_x_Se_y_ to obtain the ternary nickel-based selenide, Fe_0.2_Ni_0.8_Se (FNSs). The HER activity of FNS increased two-fold at 10 mA/cm^2^, and the overpotential of OER decreased to 255 mV at 50 mA/cm^2^. The synthetic strategy and research results of this work have a certain reference value for other low-cost and high-efficiency transition metal catalysts for electrocatalytic water splitting.

## 1. Introduction

Hydrogen from water electrolysis has been sought after for industrial applications, because of its characteristics of a high energy density and zero emissions [1,2]. The total efficiency of water electrolysis depends on two half-reactions, namely the hydrogen evolution reaction (HER) at the cathode and the oxygen evolution reaction (OER) at the anode [3,4]. The actual applied voltage values in commercial electrolysis tanks (1.8~2.0 V) are higher than the theoretical values of 1.23 V versus reversable hydrogen electrodes (RHEs), mainly due to the sluggish dynamics of the four-electron process, triggered by the high overpotential of OER [5,6]. Ru/Ir and their oxides (e.g., RuO_2_ [7], IrO_2_ [8]) are widely used as commercial catalysts because of their high reaction dynamics during HER and OER. However, limited resources and their high-cost prevent the practical application of water electrolysis. Therefore, it is vital to develop an Earth-abundant transitional metal-based (3d-based) material as a catalyst for both HER and OER [9,10,11,12,13].

In recent years, researchers have found that 3d metal-based catalysts, such as alloys [14,15], oxides [16,17], sulfides [18,19], carbides [20,21], and phosphides [22,23], exhibit high activity for both HER and OER. Among these emerge catalysts such as metallic sulfur compounds that have attracted extensive research interests due to their processability at the nanoscale, their high electrochemical activity, and their low price [24,25]. NiS-Ni_2_P_2_S_6_ nanosheets show a high catalytic activity for both HER and OER, benefiting from a unique three-dimensional self-supporting structure [26]. Stainless steel was found to be particularly suited for cathode–anode pairs in alkaline-based water electrolysis systems. In addition, NiSe nanowires on foam nickel (NF) also show high bifunctional catalytic activity in an alkaline solution [27]. These electrodes have three-dimensional structures and an ordered surface, ensuring a high exposure ratio of catalytic active sites. In this sense, the fractal-structured nanomaterials can show more unique properties than the materials with ordered surfaces. For instance, the enhanced localized field on the tips of fractal Au nanoneedles can gather ions to make a localized high-ion concertation, and thus accelerates catalytic activity [28,29,30]. Combining the enhanced field on the tip and abundant characteristics, the fractal structure shows ultra-high HER and OER activity during water splitting [31,32].

In this work, we synthesized nickel-based selenide (Ni_x_Se_y_) with a fractal structure using a solvent thermal method. By variating the aging time, the Ni_x_Se_y_ obtained at 80 min (168 °C) showed the best catalytic activity, both for HER (225 mV at 10 mA/cm^2^) and OER (309 mV at 50 mA/cm^2^). Based on this method, we synthesized the ternary transition metal selenide, M_x_Ni_y_Se_z_ (M = Fe, Mn and V), catalysts with different 3d electron numbers to achieve a local electron distribution [10,33,34,35,36]. Among these samples, Fe_0.2_Ni_0.8_Se showed the best bifunctional electrochemical activities, and the overpotential of HER and OER were 124 mV (at 10mA/cm^2^) and 255 mV (at 50 mA/cm^2^), respectively. The high catalytic activity of Fe_0.2_Ni_0.8_Se was associated with its large electrochemical surface area, the highly intrinsic activity of Fe_0.2_Ni_0.8_Se, and the abundant hetero-interfaces in the crystal. These results may shed light on the mechanisms of designing high-performance 3d metal-based catalysts.

## 2. Experimental Section

### 2.1. Chemical Reagents

Ethylene glycol (C_2_H_6_O_2_, >99%), polyvinylpyrrolidone (PVP, molecular weight 40,000), selenium powder (Se), nickel belts (NBs), VCl_3_, MnSO_4_, FeSO_4_, and Co(NO_3_)_2_·6H_2_O were purchased from Aladdin. The above chemicals were used directly without further purification.

### 2.2. Synthesis of Ni_x_Se_y_ and M_x_Ni_y_Se_z_ on NBs

Ni_x_Se_y_ on NBs: 1 g PVP and 10 mmol Se powder were dissolved into 40 mL ethylene glycol (EG). Then, the solution was heated to 168 °C with a stirring rate of 600 rpm. NBs, which were cleaned using HCl (0.01 M) (sonicated for 5 min) and ethanol, were soaked into pre-heated EG solution and held for different times. The obtained samples ere washed with water and ethanol, and then dried at 60 °C for 1 night. The NBs with surface modifications were stored in a glass bottle in an Ar atmosphere. The aging times were 40, 60, 80, and 120 min.

M_x_Ni_y_Se_z_ on NBs: 1 g PVP and 10 mmol Se powder were dissolved into 40 mL ethylene glycol (EG). Then, the solution was heated to 168 °C with a stirring rate of 600 rpm. Then, 5 mL—salt solution (0.1 M) was added to an EG solution (in these experiments, VCl_3_, MnSO_4_, and FeSO_4_, were used as the metal–salt source). After 5 min, the prepared samples were soaked in solution and held for 2 h, washed with water and ethanol, and then dried at 60 °C for 12 h. The aging temperature was kept at 168 °C for 80 min.

### 2.3. Structural Characterization

An XRD diffractometer (model: Bruker D8 Advance, Billerica, MA, USA) was used to determine the phase and crystallinity of the synthetic samples. A field emission scanning electron microscope, model Gemini SEM 300 (Jena, Germany), was used to characterize the surface morphology of Ni_x_Se_y_ and M_x_Ni_y_Se_z_. The elemental composition and chemical states (atomic valence, inner electron binding energy displacement, etc.) of Ni_x_Se_y_ and M_x_Ni_y_Se_z_ were further determined using an X-ray photoelectron spectrometer (Thermo Fisher Escalab 250Xi, Waltham, MA, USA).

### 2.4. Electrochemical Measurements

All electrochemical measurements were performed in a system configured with three electrodes that were connected to a CHI760E electrochemical workstation (Tesco Shanghai, Shanghai, China). A carbon rod was used as a counter electrode, an Ag/AgCl electrode was used as the reference electrode, and self-supported Ni_x_Se_y_ and M_x_Ni_y_Se_z_ were used as the working electrode. The electrolyte was 1.0 M KOH and preparations (e.g., cleaning the electrode surface, calibrating the reference electrode, evaluating the HER and OER properties of the sample material, and activating the electrode material) were conducted before the electrochemical experiments. In our experiments, calculation from the working electrode potential (*E_Ag/AgCl_*) to the reversible hydrogen electrode potential (*E_RHE_*) followed:

$$E_{RHE}=0.197+0.0591\times pH+E_{Ag/AgCl}$$

## 3. Results and Discussion

### 3.1. Structural Analysis of Ni_x_Se_y_

**Figure 1:**
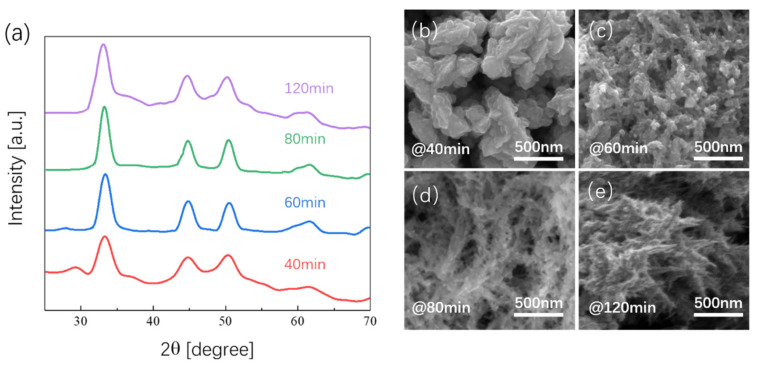
(**a**) XRD pattern of Ni_x_Se_y_ with different aging times; (**b**–**e**) SEM images of Ni_x_Se_y_ with different aging times.

The X-ray diffraction (XRD) patterns are shown in Figure 1a, including those of prepared Ni_x_Se_y_ samples with different aging times. The peak located at 27.8° belongs to the (100) face of NiSe (JCPDS#02-0892). The (100) peak vanished once the aging time increased to 80 min, indicating variations in surficial atomic arrangement during the aging process. In contrast, diffraction peaks of the Ni-based selenides at 32.8°, 44.5°, 50.0°, 59.6°, and 61.5° corresponded to the NiSe (JCPDS#02-0892) of (101), (102), (110), (103), and (201), respectively, and peaks at 29.2°, 33.6°, 44.8°, 50.6°, 60.4°, 61.9°, and 69.1° represented Ni_3_Se_4_ (103), (202), (114), (310), (116), (402), and (202) according to PDF card JCPDS#18-0890. In these XRD patterns, the characteristic peaks were highly similar, indicating that the crystal structure and compositions of NiSe were highly stable during the synthesizing process. Figure 1b–e shows SEM images of Ni_x_Se_y_ samples with different aging times. At the beginning, the Se compounds bonded to Ni atoms on NBs and formed large NiSe particles with diameters of 200–300 nm. Once the aging time increased to over 80 min, the samples showed obvious dendritic fractal structures (Figure 1d,e). The sample aging for 80 min showed a certain number of small holes on the surface. In comparison, a dendritic fractal structure is denser and there are no small holes on the surface in sample aged for 120 min. These results verify the complex three-dimensional features of fractal NiSe structures. 

**Figure 2:**
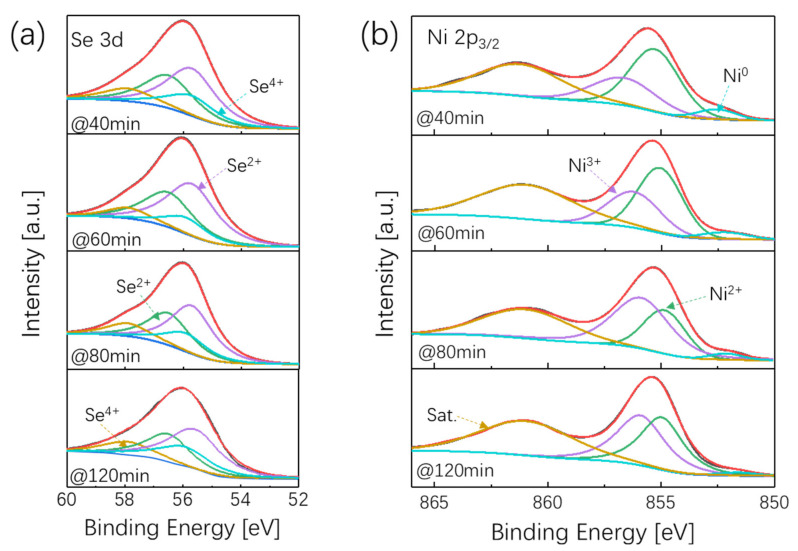
High-resolution XPS spectra of NixSey with different aging times. (**a**) Se 3d; (**b**) N 2p.

In order to compare the electronic structure of prepared samples under different aging times, X-ray photoelectron spectroscopy (XPS) measurements of the Ni_x_Se_y_ nanostructures were performed and the results are shown in Figure 2a,b. As demonstrated in Figure 2a, the peaks located at 56.1 eV and 58 eV corresponded to Se^4+^, and the peaks located at 55.6 eV and 56.5 eV corresponded to Se^2+^. There was no distinct impact on the valence state of Se when changing the aging time of the Ni_x_Se_y_ samples. Figure 2b shows the Ni 2p XPS spectra of samples with different aging time. The peak around 861.3 eV is the satellite peak in the prepared samples, and the peak of Ni 2p could be resolved into two types. The binding energy peak at ≈856.8 eV belongs to Ni^3+^ 2p_3/2_ and the lower energy peak at ≈855.3 eV represents Ni^2+^ 2p_3/2_. Obviously, the aging time exhibited a significant influence on the orbital occupancy of the Ni cation. With the aging time increasing from 40 min to 120 min, the proportion of Ni^3+^ bands in the Ni_x_Se_y_ samples first increased and then decreased; the peak of Ni^3+^ reached its maximum when the aging time was 8 min. As it is known that Ni ions with a high valence are the main active sites in electrochemical reactions [30], the electronic structure of Ni_x_Se_y_ samples that we tested were closely related to the subsequent electrochemical results.

### 3.2. Electrocatalytic Performance of Ni_x_Se_y_

**Figure 3:**
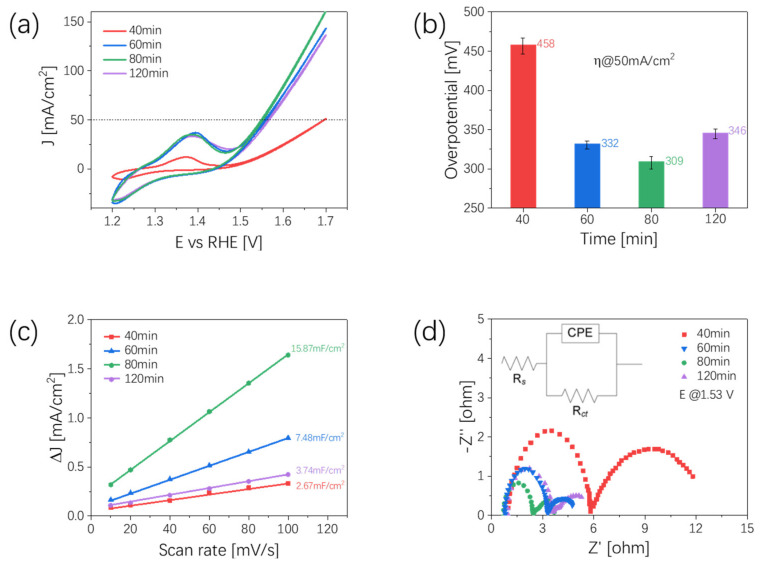
Oxygen evolution reaction characterizations of Ni_x_Se_y_ at different aging times. (**a**) CV curves of Ni_x_Se_y_ after iR-correction; (**b**) relationship between OER overpotential at 50 mA/cm^2^ and aging time; (**c**) C_dl_ values and relative electrochemical active surface areas; (**d**) Nyquist plots at 1.53 V vs. RHE, the inset image shows the equivalent circuit.

The electrochemical activities of OER of Ni_x_Se_y_ under different aging times were assessed in a 1.0 M KOH electrolyte. Figure 3a shows the cycle voltammetry (CV) curves for Ni_x_Se_y_ under different aging times, ranging from 40 min to 120 min. It can be seen that, at a current density of 50 mA/cm^2^, the sample aged for 80 min showed the lowest overpotential of around 309 mV. Once the aging time reached 120 min, the overpotential reached a higher value of 346 mV. Comparatively, the overpotentials of the Ni_x_Se_y_ samples under aging times of 40 min and 60 min were 458 mV and 332 mV, respectively (Figure 3b). To determine the electrochemically active surface area (ECSA) of Ni_x_Se_y_, we tested C_dl_ by measuring the non-Faradaic capacitive current associated with double-layer charging from the scan-rate dependence of the cyclic voltammograms. According to the formula ECSA = C_dl_/C_s_, where C_dl_ is the electrochemical double-layer capacitance and C_s_ is the specific capacitance of the sample or the capacitance of an atomically smooth planar surface of a material per unit area under identical electrolyte conditions [33]. The voltammograms of the Ni_x_Se_y_ samples were tested at different scan rates, from 60 to 180 mV/s, in a voltage range of 1.1 to 1.2 V (vs. RHE). As shown in Figure 3c, the sample with an aging time of 80 min possessed a remarkably larger C_dl_ value (15.87 mF/cm^2^) than that of the 40 min (2.67 mF/cm^2^), 60 min (7.48 mF/cm^2^), and 120 min (3.74 mF/cm^2^) samples, considering that the C_s_ of as prepared Ni_x_Se_y_ sample was the same, which means there are more active sites involved in the electrochemical reaction in the 80 min sample and the promoted OER performance of the 80 min sample could be attributed to its relatively larger ECSA. The ECSA results were also consistent with the XPS results in Figure 2b, indicating that when the aging time was 80 min, the number of electrochemically active sites in Ni_x_Se_y_ was the largest. To better understand the electrode kinetics during OER, electrochemical impedance spectroscopy (EIS) was also performed. Nyquist plots were fitted using an equivalent circuit, where a low charge transfer resistance (*R_ct_*) is beneficial to the transfer of electrons in electrocatalytic reactions. As is shown in Figure 3d, the *R_ct_* values of Ni_x_Se_y_ samples for 40 min, 60 min, 80 min, and 120 min were 5.82 Ω, 3.29 Ω, 2.43 Ω, and 3.67 Ω, respectively. The relatively lower *R_ct_* of the 80 min sample illustrates its faster electron transfer and desirable electrocatalytic kinetics.

**Figure 4:**
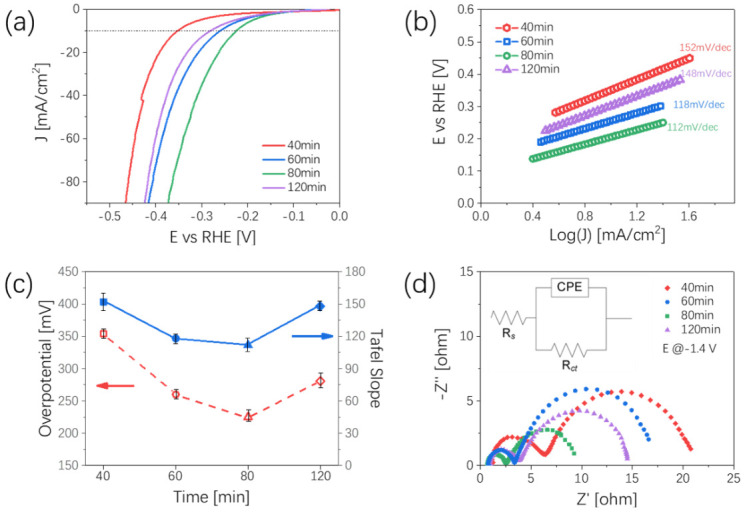
Hydrogen evolution reaction characterizations of Ni_x_Se_y_ with different aging times. (**a**) LSV curves of Ni_x_Se_y_ after iR-correction; (**b**) Tafel slope images; (**c**) relationship between Tafel slope and overpotential at 10 mA/cm^2^ at different aging times; (**d**) Nyquist plots at −1.4 V vs. RHE, the inset image shows the equivalent circuit.

Under the same electrolyte conditions, we also tested the HER activity of Ni_x_Se_y_ samples with different aging times. The results of linear sweep voltammetry (LSV) curves are shown in Figure 4a. The variation trend of HER activity with aging time is the same as that of the OER results. The 80 min sample exhibited a significantly improved HER activity, displaying a low overpotential of 225 mV to drive a current density of 10 mA/cm^2^, which was smaller than those of 40 min (354 mV), 60 min (260 mV), and 120 min (281 mV). Then, we tested the Tafel slope of the Ni_x_Se_y_ samples with different aging times, shown in Figure 4b; the Tafel slope of the 40 min, 60 min, 80 min, and 120 min samples were 152 mV/dec, 118 mV/dec, 112 mV/dec, and 148 mV/dec, respectively. Samples with a 80 min aging time obtained the lowest Tafel slope, which meant faster electrochemical reaction kinetics. Meanwhile, we summarized the overpotential and the Tafel slopes of Ni_x_Se_y_ samples by changing the aging time, and found that the values of such results maintained the same variation trends (Figure 4c). Electrochemical impedance spectroscopy results are shown in Figure 4d. Not surprisingly, the 80 min samples possessed the lowest *R_ct_* value (2.49 Ω), and the *R_ct_* values of samples at 40 min, 60 min, and 120 min aging times were 6.25 Ω, 3.44 Ω, and 3.95 Ω, respectively.

Here, we prepared different Ni_x_Se_y_ samples by changing the aging time. Electrochemical tests results show that the electrochemical activity of Ni_x_Se_y_ does not change linearly with an increase in aging time. However, when the aging time was maintained at 80 min, Ni_x_Se_y_ exhibited the best electrochemical activities, whether in the OER or HER process. Through the XPS results and electrochemical characterization of Ni_x_Se_y_ samples at different aging times, we found that this performance is the result of the comprehensive effects of many aspects, including the number of active sites in the electrochemical reaction, the valence of Ni, and the electrochemical impedance of the samples. In summary, when the aging time was 80 min, the proportion of Ni^3+^ in the Ni_x_Se_y_ samples was the largest, which had a positive effect on the OER performance. At the same time, the electrochemically active surface area of the sample reached its maximum under this condition, which indicated that the sample had the strongest electrochemical activity at that time. Through the EIS test, it was found that the electrochemical impedance of the sample reached its minimum in either the HER or OER test environment, which indicated that the electron transport efficiency of the sample participating in the electrochemical reaction was the highest at that time. These results are reasons why the electrochemical performance of the Ni_x_Se_y_ sample was the best when the aging time was 80 min.

### 3.3. Electrocatalytic Performance of M_x_Ni_y_Se_z_

**Figure 5:**
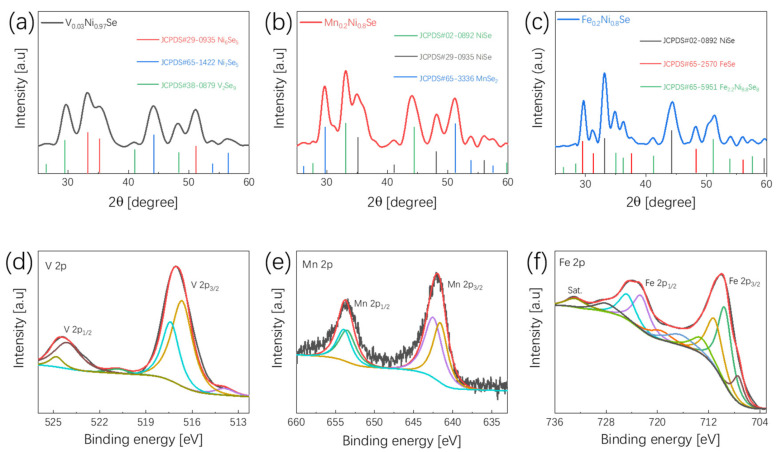
XRD patterns of (**a**) V_0.03_Ni_0.97_Se; (**b**) Mn_0.2_Ni_0.8_Se; and (**c**) Fe_0.2_Ni_0.8_Se. High-resolution XPS spectra of (**d**) V 2p; (**e**) Mn 2p; (**f**) Fe 2p.

In the above section, we explained the experimental conditions for Ni_x_Se_y_ to exhibit the best electrochemical performance (maintaining the aging time at 80 min in our present experiment). In order to explore more efficient bifunctional water splitting catalysts, we used the same method (the experimental conditions were kept constant, and the aging time was 80 min) to synthesize M_x_Ni_y_Se_z_ as bifunctional catalysts, where M represents V, Mn, and Fe. The XRD results are shown in Figure 5a–c, it can be seen from the PDF cards that there are different kinds of diffraction peaks. We can conclude that the chemical formulas of our prepared samples are V_0.03_Ni_0.97_Se (VNS), Mn_0.2_Ni_0.8_Se (MNS), and Fe_0.2_Ni_0.8_Se (FNS), and the XPS results of the 2p orbital of these transition metals are shown in Figure 5d–f. In Figure 5d, the peaks of V 2p_3/2_ and 2p_1/2_ are located at 517.1 eV and 524.4 eV. While in Figure 5e, the peaks at 642.4 eV and 653.9 eV represent Mn^4+^, the peaks at 641.5 eV and 653.4 eV represent Mn^2+^ in the prepared MNS samples. Figure 5f shows the XPS result of the Fe in the FNS samples, the peaks in the binding energy of 711.1 eV and 724.7 eV are Fe^3+^ and the peaks in the binding energy of 709.6 eV and 722.5 eV are Fe^2+^.

**Figure 6:**
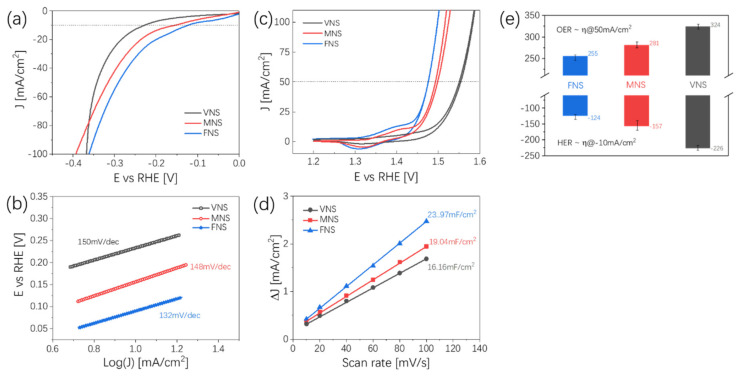
Electrochemical performance of MNi_x_Se_y_ (M = V, Mn and Fe). (**a**) LSV curves of samples in HER; (**b**) Tafel slope results of HER; (**c**) CV curves of samples in OER; (**d**) C_dl_ values and relative electrochemical active surface areas in the OER process; (**e**) comparison of HER (overpotential @ 10mA/cm^2^) and OER activities (overpotential @ 50 mA/cm^2^).

The catalytic HER performance of Ni_x_Se_y_ doped with different transition metals is shown in Figure 6. We kept the scan rate at a low value (5 mV/s) to ensure a minimum capacitive current. As demonstrated in Figure 6a, FNS exhibited the best HER activity with the lowest overpotential of 124 mV at a current density of 10 mA/cm^2^. Compared with Ni_x_Se_y_ (225 mV), the addition of Fe increased the HER activity of the prepared sample by nearly two times with a current density of 10 mA/cm^2^. The overpotential of the FNS was also lower than that of VNS (226 mV) and MNS (157 mV). Tafel slopes are shown in Figure 6b; the Tafel slope results of the four different samples are basically at the same level, where FNS has the lowest Tafel slope of 132 mV/dec and VNS has the highest Tafel slope of 150 mV/dec. The similar Tafel slopes indicated that the electrochemical kinetics of these different samples are basically the same during the HER process, while different electrochemical performances may be due to the intrinsic activity of different elements on HER. Figure 6c shows the OER property of the ternary nickel-based selenide using cycle voltammetry measurement, it can be clearly seen from the figure that FNS has the best OER performance among those samples, with an overpotential of 255 mV at a current density of 50 mA/cm^2^, and 54 mV lower overpotential compared to Ni_x_Se_y_. The change trend in the OER activity was similar to that of HER, the specific values are shown in Figure 6e. Therefore, we can conclude that when doped with different kinds of transition metals (V, Mn, Fe) in Ni_x_Se_y_, the activities of HER and OER increased on the order of VNS < MNS < FNS. The OER electrochemical active surface area results (expressed in C_dl_ value) of those samples are also shown in Figure 6d; the reason why FNS yielded the best OER activity among those ternary nickel-based selenide is that it possesses the largest C_dl_ (23.97 mF/cm^2^) among these materials, including the C_dl_ of MNS (19.04 mF/cm^2^) and VNS (16.16 mF/cm^2^). This enables more active sites to be involved in the catalytic process in FNS.

**Figure 7:**
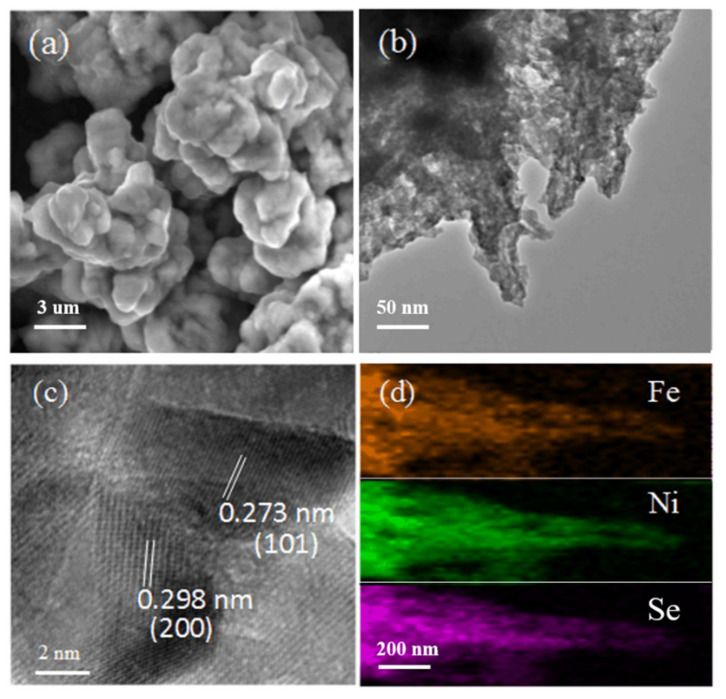
SEM and TEM characterization maps of Fe_0.2_Ni_0.8_Se. (**a**) Fe_0.2_Ni_0.8_Se—low magnification SEM; (**b**) Fe_0.2_Ni_0.8_Se—low magnification TEM; (**c**) Fe_0.2_Ni_0.8_Se—high magnification TEM; (**d**) EDS mapping of Fe, Ni, Se.

Then, we tested the SEM and TEM results of the FNS samples. Figure 7a shows an SEM image of FNS, and it can be seen that the structure of the FNS is transformed from its original fractal structure into a three-dimensional particle stack. Figure 7b shows a TEM image of the FNS at a low magnification. It can be observed that the FNS structure still has a small branching structure in some regions. Further observation of Figure 7c shows that the analysis yields that FNS has two lattice gaps, one at 0.272 nm and another at 0.298 nm. From the Bragg diffraction formula, the diffraction peak index can be transformed into the <101> and <200> crystallographic directions, which means that the growth direction, as well as the growth axis of the sample, are in the <101> crystallographic direction and <200> crystallographic direction, where the <101> crystallographic direction is consistent with the crystallographic direction of NiSe and the <200> crystallographic direction is consistent with the crystallographic direction of FeSe_2_. Figure 7d shows the EDS mapping diagrams of Fe, Ni, and Se, respectively. Orange corresponds to iron, green corresponds to nickel, and pink corresponds to selenium, which show the even distribution of iron, nickel, and selenium elements.

## 4. Conclusions

In conclusion, an efficient bifunctional water splitting catalyst composed of Ni_x_Se_y_ was fabricated using a simple method. We conclude that, when keeping the aging time at 80 min, the catalyst has the best catalytic activities with an HER overpotential of 225 mV (η@10 mA/cm^2^) and an OER overpotential of 309 mV (η@50 mA/cm^2^). Furthermore, when we doped Fe into Ni_x_Se_y_ under the same experimental conditions to synthesize ternary nickel-based selenide (Fe_0.2_Ni_0.8_Se), the catalytic performance was significantly improved, with an overpotential of 124 mV (η@10 mA/cm^2^) for HER and 255 mV (η@50 mA/cm^2^) for OER. This work explored the best experimental conditions for obtaining the most active Ni_x_Se_y_ for electrocatalytic water splitting, and the electrochemical performance was optimized by doping different transition metals. This may provide a brand-new idea for future synthesis and research based on transition metal catalysts.

## Data Availability

Not applicable.
